# Application of Antimicrobial Peptides (AMPs) in Treatment of Osteomyelitis in Human and Veterinary Orthopedics

**DOI:** 10.3390/jfb16030090

**Published:** 2025-03-05

**Authors:** Dominika Nguyen Ngoc, Michał Latalski, Anna Danielewicz, Tomasz Szponder, Joanna Wessely-Szponder, Ewa Mazur

**Affiliations:** 1 Sub-Department of Pathophysiology, Department of Preclinical Veterinary Sciences, Faculty of Veterinary Medicine, University of Life Sciences, 20-033 Lublin, Poland; dominika.nguyen@up.lublin.pl (D.N.N.); ewa.mazur@up.lublin.pl (E.M.); 2 Children’s Orthopaedic Department, Medical University of Lublin, 20-093 Lublin, Poland; michal.latalski@umlub.pl (M.L.);; 3 Department of Diagnostics and Clinical Sciences, Faculty of Veterinary Medicine, University of Agriculture in Krakow, 31-120 Cracow, Poland

**Keywords:** osteomyelitis, antimicrobial peptides (AMPs), bone regeneration, biomaterials, bone infection

## Abstract

Osteomyelitis, a severe bone infection, poses a significant therapeutic challenge in both human and veterinary medicine, especially due to the increasing prevalence of antibiotic-resistant pathogens like methicillin-resistant Staphylococcus aureus (MRSA). Conventional treatments, including surgical debridement and systemic antibiotics, often prove inadequate due to the ability of bacteria to form biofilms and evade host immune responses. Antimicrobial peptides (AMPs), such as LL-37 and β-defensins, have emerged as a promising alternative therapeutic strategy. AMPs exhibit broad-spectrum antimicrobial activity, including efficacy against resistant strains, and possess immunomodulatory properties that can promote bone regeneration. This article comprehensively reviews AMP applications in treating osteomyelitis across both human and veterinary medicine. We discuss diverse therapeutic approaches, including free AMPs, their conjugation with biomaterials such as collagen and chitosan to enhance delivery and stability, and the development of AMP-based nanoparticles. Furthermore, we analyze preclinical and clinical findings, highlighting the efficacy and safety of AMPs in combating osteomyelitis in both human and animal patients. Finally, we explore future perspectives and challenges, such as optimizing delivery, stability, and efficacy, while minimizing cytotoxicity, and in translating AMP-based therapies into clinical practice to effectively manage this debilitating disease.

## 1. Introduction

Osteomyelitis, a severe infection affecting the bone and bone marrow, poses a significant challenge to human and veterinary healthcare systems. This debilitating condition places a substantial burden on patients and healthcare resources alike. In human medicine, osteomyelitis is a growing concern in orthopedics, particularly after surgical interventions such as implant insertion for fracture fixation or joint replacement [1]. Similarly, osteomyelitis is a frequent complication in veterinary medicine, affecting various animal species [2].

Characterized by inflammation, bone destruction, and the formation of necrotic tissue, osteomyelitis manifests through a range of symptoms, including pain, swelling, purulent discharge, and local hyperthermia [1,3,4]. The infection can arise from various sources, including direct contamination through open wounds, the spread of infection from adjacent soft tissues, or hematogenous dissemination via the bloodstream [5].

This review provides a comprehensive overview of osteomyelitis, exploring its impact on human and veterinary medicine. We delve into the role of antimicrobial peptides (AMPs) as a promising therapeutic strategy for combating this challenging condition. Furthermore, we discuss AMP-based therapies’ challenges and future perspectives in human and veterinary orthopedics.

## 2. General Overview: Definition, Classification, and Pathogenesis of Osteomyelitis

### 2.1. Definition and Classification of Osteomyelitis

Osteomyelitis, or osteitis, is an inflammatory process affecting the bone, encompassing the haversian spaces, Volkmann canals, medullary cavity, and periosteum. This type of infection commonly develops in cases of open fractures, bone surgeries (particularly those involving metallic implants), and systemic diseases [2]. Osteomyelitis is characterized by an inflammatory reaction and bone destruction resulting from bacterial colonization of the bone, bone marrow, and surrounding tissues [5,6]. These infections are painful for patients and pose significant challenges for healthcare providers. Unlike many other infectious diseases that respond well to antimicrobial therapy, osteomyelitis treatment is often complicated by bone tissue’s unique structural and functional properties. Early diagnosis, including bone sampling for microbiological and histological examination, is crucial for guiding targeted and long-term antimicrobial therapy [6].

According to Lew et al., osteomyelitis can be classified into three main categories [6], including the following.

1. Hematogenous Osteomyelitis: This category refers to bone infections that originate from the bloodstream [7]. Bacteria circulating in the blood seed the bone tissue, typically affecting the metaphysis of long bones in children or the vertebral bodies in individuals of all ages [1].
2. Osteomyelitis due to Contiguous Spread (without Vascular Insufficiency): This category encompasses infections that spread directly from a nearby source, often following trauma or surgical intervention. Bacteria can enter the bone through direct inoculation (e.g., an infected fracture) or by extending from contaminated soft tissues (e.g., a prosthetic joint infected during implantation) [7].
3. Osteomyelitis resulting from Contiguous Spread (with Vascular Insufficiency): This category predominantly occurs in the lower extremities and is often linked to diabetic foot infections [6,7]. Poor blood supply, diabetic wounds, and impaired immune responses contribute to the development of osteomyelitis in this setting [1].

In each of these categories, osteomyelitis may develop as either an acute or chronic condition, with various bacteria—and occasionally fungi—being responsible for the infection. Therefore, the management of osteomyelitis must be individualized, considering each case’s specific circumstances [7].

### 2.2. Etiological Factors in Human Patients

Although various microorganisms can cause osteomyelitis, pyogenic bacteria, particularly *S. aureus*, are the most common culprits [1]. According to Figuereido et al. *S. aureus* is identified in above 60% of isolates and together with Coagulase-negative Staphylococcus (CoNS), such as *S. epidermidis* (29.9%), *S. lugdunensis* (5.2%), *S. haemolyticus* (1.5%), *S.warneri* (1.3%), and, *S. hominis* (1.3%), is a causative factor for up to two-thirds of all osteomyelitis and septic arthritis [8]. Other pathogens are among Streptococcus spp. and aerobic Gram-negative bacilli [9,10].

**Figure 1:**
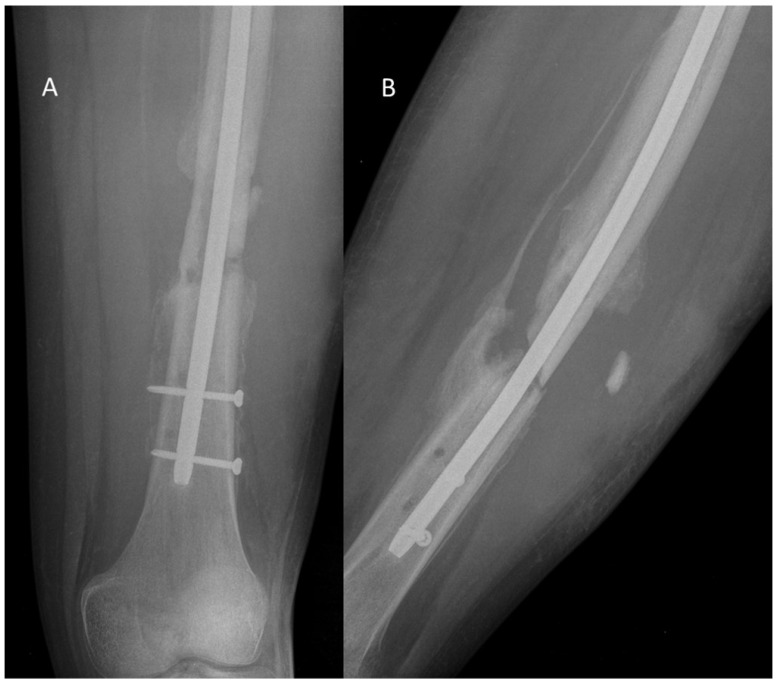
Radiographs of a 17-year-old patient with chronic osteomyelitis of the femur due to infection of an intramedullary nail. The anteroposterior (**A**) and lateral (**B**) projections show the presence of osteolytic changes, periosteal reaction, and periosteal calcifications, suggesting a chronic course of infection. Features of bone destruction and lack of proper bone union are visible, which may indicate infection with antibiotic-resistant microorganisms.

*S. aureus* produces multiple virulence factors that play a key role in the onset and persistence of osteomyelitis. For instance, adhesins facilitate attachment to the extracellular matrix of bone, primarily fibronectin and collagen. After colonizing bones, *S. aureus* can establish a chronic infection by surviving within abscesses and the canalicular network, where it remains protected from antimicrobials and the immune system. It can also adhere to bone sequestration, making infection elimination more difficult. Biofilm formation on implanted materials or necrotic bones is another key mechanism for *S. aureus* persistence in osteomyelitis (Figure 1) [1].

### 2.3. Pathogenesis

**Figure 2:**
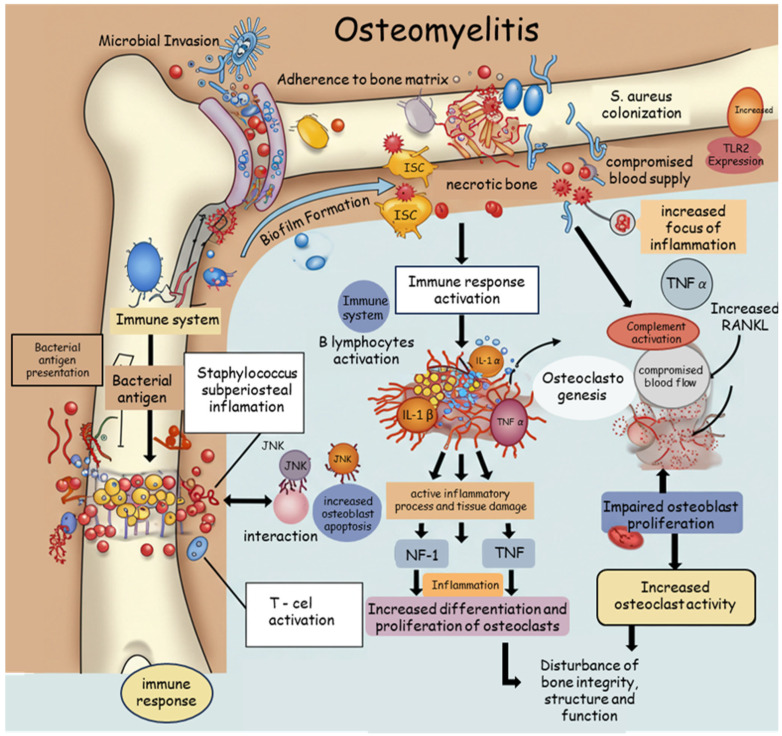
Pathophysiology of osteomyelitis. Bacterial infection, primarily caused by *S. aureus*, leads to microbial adhesion to the bone matrix, biofilm formation, and bone colonization. This triggers activation of the immune system, including B lymphocytes and T cells, resulting in an enhanced inflammatory response. Pro-inflammatory cytokines such as IL-1β and TNF-α stimulate osteoclastogenesis, increasing bone resorption. Compromised blood supply and complement activation contribute to impaired osteoblast proliferation and increased osteoclast activity, ultimately leading to bone tissue destruction and loss of structural integrity.

The pathogenesis of osteomyelitis involves a complex interplay between microbial invasion, host immune responses, and the unique characteristics of bone tissue. This multifaceted process can be divided into three key stages: bacterial invasion and biofilm formation, the immune response to the biofilm, and the effects of bacterial infiltration on bone tissue components (Figure 2) [11].

#### 2.3.1. Microbial Invasion and Biofilm Proliferation

Once bacteria enter the bone tissue, they trigger an acute inflammatory response. This inflammation, along with bacterial activity, impacts the periosteum and spreads throughout the bone, leading to necrosis. In children, the loosely attached periosteum facilitates the formation of subperiosteal abscesses. These further compromise the blood supply, resulting in segmental bone necrosis (sequestrum). In the chronic stage, different inflammatory cells and their release of cytokines stimulate osteoclastic bone resorption, ingrowth of fibrous tissue, and reactive new bone deposition (involucrum). The rupture of subperiosteal abscesses can lead to soft-tissue abscesses and draining sinuses [1]. It is worth noting that studies using experimental models, which require a high bacterial inoculum to induce osteomyelitis, have confirmed that healthy bone exhibits strong resistance to infection [7].

#### 2.3.2. Immune Response to Bacterial Biofilm

The host immune system mounts a vigorous response to bacterial invasion, but this response can also contribute to bone injury. Several cytokines exhibit osteolytic properties, while phagocytes generate harmful reactive oxygen species (ROS) and proteolytic enzymes that can damage host cells [12]. The inflammatory response increases intraosseous pressure, reducing blood flow and leading to ischemic necrosis. The resulting necrotic bone (sequestrum) serves as a platform for biofilm formation, enabling bacteria to enter a low-metabolic state and persist in a low-oxygen microenvironment [13]. Reduced blood circulation and biofilm formation hinder the ability of antimicrobial agents and immune cells to reach and eliminate the bacteria [7].

It is well established that opsonization of planktonic bacteria by immunoglobulins and complement triggers neutrophil activation, leading to enhanced phagocytosis and ROS production. Neutrophils also target bacterial biofilms; however, their effectiveness depends on the biofilm’s maturation stage. Due to lower clearance efficiency, immature biofilm structures are more susceptible to neutrophil attack [11,14,15].

Circulating monocytes might significantly contribute to bone resorption in inflammatory bone disorders. These cells showed a higher rate of cellular adhesion at the vascular endothelium and transendothelial migration, which resulted in enhanced osteoclast differentiation when stimulated by tumor necrosis factor (TNF)-α and interleukin (IL-1) [11]. Moreover, an increased expression of macrophage inflammatory proteins (MIP1α, CCL3) and MIP2α (CXCL2) were found in bone samples of patients suffering from an infection of orthopedic prostheses. When stimulated by bacteria, osteoblasts could also produce macrophage inflammatory proteins [11,16].

#### 2.3.3. Impact of Bacterial Invasion on Bone Tissue Components

Generally, healthy bone tissue is inherently infection resistant. Therefore, clinical osteomyelitis often develops in the presence of concurrent soft tissue injury, compromised vascularity, sequestration, implants, or immune deficits. While bacterial contamination is common during surgery (72% in open fractures, 39% in closed fractures), clinical infection occurs in fewer cases. Metallic implants can depress host defense mechanisms and promote infection by fostering bacterial biofilm formation, which protects bacteria from immune responses and enhances their adherence to implants [2,17].

Infection in bone triggers a cascade of events, including vascular congestion, edema, and inflammatory exudate, leading to bone cell death and the spread of infection along the medullary canal and periosteum. Increased intraosseous pressure and reduced blood flow contribute to tissue necrosis. During the healing phase, pyogenic granulation tissue removes dead bone and segregates necrotic areas as sequestra, while the periosteum forms new bone (involucrum) around the infected region. Pus may drain through sinus tracts, causing scarring and skin thinning. Fracture instability further exacerbates the condition by preventing revascularization. Timely surgical or medical intervention can halt the infection, enabling gradual bone remodeling into a structure resembling the original bone [2,18].

Bacterial presence directly affects the cellular components of bone tissue. Infection with *S. aureus* leads to increased Toll-like receptor 2 (TLR2) expression as part of the innate immune system. Apoptotic cell death was further induced, and mitogen-activated protein kinase pathways were activated in osteoblasts. The expression of TLR2 and the activity level of Jun N-terminal kinases (JNK) were directly correlated to directly impact osteoblast apoptosis and osteogenic differentiation after bacterial invasion. Bacterial endotoxins, particularly lipopolysaccharides from the outer membrane of Gram-negative bacteria, can induce apoptosis and suppress osteoblast differentiation by activating the JNK pathway [11,19,20].

Another virulence factor, protein A (SpA) from *S. aureus*, can directly bind to osteoblasts, inhibiting proliferation and mineralization while simultaneously triggering apoptosis. SpA can interact with tumor necrosis factor receptor 1 (TNFR-1), which is abundantly expressed on osteoblasts. This interaction activates the NF-κB pathway, leading to the release of various cytokines, particularly IL-6, which is known to enhance osteoclast activity [11,21,22].

Cytokines belonging to the TNF superfamily are known to induce apoptotic signaling in various cell types. The TNF-related apoptosis-inducing ligand (TRAIL) can bind to receptors that trigger apoptosis, including death receptors and osteoprotegerin (OPG). OPG serves as a soluble decoy receptor for TRAIL and RANKL, both of which drive osteoclastogenesis. However, infected osteoblasts suppress OPG production, further contributing to bone degradation [11,20].

## 3. Osteomyelitis as a Problem in Veterinary Medicine

### 3.1. Introduction and Affected Species

**Figure 3:**
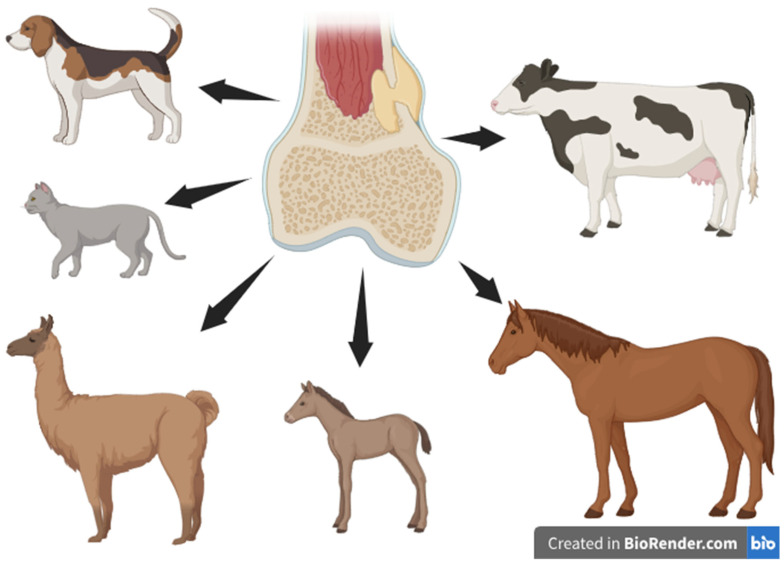
Osteomyelitis as a problem in veterinary medicine—susceptible species. Osteomyelitis is a serious condition affecting multiple animal species, including dogs, cats, cattle, horses, alpacas, and foals. This pathology can result from bacterial infections, trauma, or postoperative complications.

Osteomyelitis, an inflammation of the bone and bone marrow, is a severe condition affecting various animal species, often resulting from prior or persistent infections caused by pyogenic microorganisms. In animals, osteomyelitis is commonly caused by spontaneous or iatrogenic inoculation of pathogens into injured tissues after trauma and/or during implantation of foreign material such as implants or prostheses, and less frequently, contrary to human patients, during the haematogenous inoculation of bone. Hematogenous osteomyelitis is more common in young animals than in adults, particularly in those with predisposing factors such as inadequate passive transfer of maternal antibodies or concurrent infections leading to septicemia or septic omphalitis [23,24,25,26,27,28]. Osteomyelitis has been found in various animal species, including dogs and cats, horses, cattle, and camelids (Figure 3).

#### 3.1.1. Dogs and Cats

The most common causes of osteomyelitis in companion animals were described as involved in trauma (post-traumatic) and as infections after surgery (post-operative). Injuries from fights or trauma caused by collisions with motor vehicles can lead to direct bone contamination through penetrating wounds or open fractures, often accompanied by damage to the surrounding vasculature. Post-traumatic osteomyelitis has also been reported in cats. In adult dogs, hematogenous osteomyelitis is rare and typically presents with lameness and/or abscess formation, often without a history of prior injury [28,29].

#### 3.1.2. Horses

Osteomyelitis is relatively common in horses, with hematogenous osteomyelitis occurring more frequently in foals, particularly in neonates rather than in adults. This increased susceptibility is often due to bacteremia, which may arise from conditions such as omphalitis or inadequate passive transfer of maternal immunity, leading to a weak response to infection. The infection typically localizes near the physis, affecting the metaphysis or epiphysis and potentially resulting in septic epiphysitis, metaphysitis, physitis, or septic arthritis. In older horses, some studies have also reported cases of post-traumatic osteomyelitis [24,30].

#### 3.1.3. Cattle

In cattle, osteomyelitis is mostly caused by *Trueperella pyogenes*. In addition to haematogenous forms in neonates, trauma associated forms were also reported. In cows, post-traumatic osteomyelitis can be caused by penetrating injuries or decubitus ulcers on the distal limb. However, hematogenous osteomyelitis was described as more common than post-traumatic cases, with affected carpus, metacarpus, tarsus, and metatarsus. [23,24].

#### 3.1.4. Camelids

In alpacas and llamas, most reported cases of osteomyelitis are either traumatic or post-operative. Some cases of haematogenous osteomyelitis were also reported. Some researchers have proposed that hematogenous osteomyelitis may develop in adult camelids, even in the absence of underlying health conditions [24,31].

### 3.2. Etiology

In veterinary medicine, osteomyelitis predominantly involves bacterial infections, although fungi and viruses can infect bone and bone marrow [2,23]. Staphylococcus species are the most common culprits, accounting for 50% to 60% of dog bone infections. Although *S. aureus* has historically been the most frequently reported pathogen, recent studies indicate that *Staphylococcus intermedius* is more prevalent in veterinary medicine, with many strains exhibiting resistance to penicillin due to beta-lactamase production [26]. However, in humans, these infections are rare and concerns mostly immunodeficient patients [32]. More recent studies point to Staphylococcus pseudointermedius as the most frequently detected pathogen in animals [27].

The rise in methicillin-resistant *Staphylococcus aureus* (MRSA) poses an increasing challenge in both human and veterinary medicine. Effective infection control measures, including personal hygiene, patient isolation protocols, and environmental decontamination, are crucial to mitigate the spread of these resistant pathogens [23,33,34].

Other bacteria commonly implicated in osteomyelitis in veterinary medicine include Streptococcus, *Escherichia coli*, Proteus, Klebsiella, Pseudomonas, and Pasteurella [2,23,35]. These organisms are often found in polymicrobial infections, particularly those arising from bite wounds. Anaerobic bacteria, such as Actinomyces, Clostridium, Peptostreptococcus, Bacteroides, and Fusobacterium, also play a significant role, especially in bite-wound-associated osteomyelitis. Studies have reported a 64% incidence of anaerobic bacteria in such cases [2,28,35]. Although less common, Fungal osteomyelitis can be caused by Cryptococcus, Candida, Blastomyces, Aspergillus, and Penicillium. The tick-borne protozoan *Hepatozoon canis* has also been identified as a cause of osteomyelitis in dogs [23]. Silveira et al. described a case of recurrent urinary tract infections in a dog linked to osteomyelitis of the penile bone. Considering this condition, they recommended the differential diagnosis of urethral obstruction in dogs with recurrent urinary tract infections [36].

### 3.3. Routes of Infection

The primary routes of infection in veterinary osteomyelitis, listed in order of frequency, are as follows:

- Direct contamination occurs through open fractures, surgical procedures for fracture treatment, and puncture wounds. Most infected fractures arise following open fracture repair or surgical intervention for closed fractures. A metallic implant is commonly associated with these infections [2].
- Direct extension from adjacent infected soft tissue: Infection can spread directly from nearby soft tissue infections to the bone [2].
- Hematogenous spread: Infection may reach the bone through the bloodstream, originating from conditions such as vertebral osteomyelitis, discospondylitis (caused by pathogens like Brucella, Nocardia, and Staphylococcus), and bacterial endocarditis. However, this route is less common than the others [2].

Osteomyelitis continues to be a major concern across various animal species in veterinary medicine. Hematogenous osteomyelitis is more frequently observed in young animals, whereas post-traumatic or postoperative cases are more prevalent in adults. A definitive diagnosis requires a thorough assessment, including clinical signs, laboratory testing, diagnostic imaging, and local biopsies for bacterial identification [28]. The primary treatment for osteomyelitis involves surgical debridement and antibiotic therapy, with implant removal when feasible. While novel antimicrobial strategies are being developed in basic science and human medicine, their adoption in veterinary practice is progressing slowly. Although managing osteomyelitis can be difficult in certain cases, ongoing advancements provide optimism for better treatment outcomes in the future [23,24,37].

## 4. Current Treatment Concepts

Since osteomyelitis often results in chronic infection with periods of remission rather than a complete cure, it is paramount to stimulate the patient’s immune system to enhance their natural defenses and promote healing. Immunostimulation can be a valuable adjunct to antibiotic therapy, bolstering the body’s ability to fight the infection [38,39]. This approach encompasses using immunomodulatory agents, ensuring proper nutrition to support immune function, and potentially incorporating adjunct therapies such as hyperbaric oxygen therapy [40]. Early and aggressive antibiotic intervention, coupled with robust immune support, is essential for effectively managing the infection and preventing recurrence [41].

The treatment of osteomyelitis is multifaceted and hinges on various clinical factors. It typically involves prolonged antimicrobial therapy and surgical interventions. The most effective approach is early and intensive treatment, although the possibility of infection recurrence months or even years later underscores the chronic nature of this condition. Current treatment modalities are not without their limitations. Antibiotic therapy carries risks such as systemic toxicity and the potential for developing antimicrobial resistance. Surgical interventions, particularly radical debridement to remove infected tissue, can lead to loss of function in the affected area. The diagnostic and therapeutic challenges associated with osteomyelitis and its detrimental impact on patient’s quality of life highlight the urgent need for more effective treatment strategies. Numerous in vitro and in vivo studies are focused on unraveling the complexities of osteomyelitis pathophysiology and developing improved diagnostic and therapeutic approaches [37,42].

Understanding the complex pathogenesis of inflammatory bone disorders is essential for developing effective treatments. This requires a comprehensive approach that considers the interactions between pathogens, the host immune system, and the cellular components of bone tissue. Although the term “osteomyelitis” might appear simple, it encompasses a complex spectrum of inflammatory bone disorders influenced by many factors. Deciphering these complexities remains a challenge for future research endeavors [11].

Current treatment paradigms primarily revolve around the surgical removal of infected tissue and aggressive antibiotic treatment to minimize the bacterial burden [5]. However, the limitations of these approaches underscore the need for innovative therapeutic strategies, such as those based on AMPs.

## 5. The Role of AMPs in Immune Response to Bone Infection

Antimicrobial peptides (AMPs), also referred to as host defense peptides (HDPs), play a crucial role in the innate immune system. In mammals, the two main classes of AMPs are cathelicidins and defensins [43]. Various cell types, including epithelial and immune cells, produce the human cathelicidin LL-37, a prominent member of this family. LL-37 exhibits diverse functions, including direct antimicrobial activity, immunomodulatory effects, and wound-healing properties. It can act as a chemoattractant for immune cells, modulate inflammation, promote angiogenesis (blood vessel formation), and even exhibit pro- and anti-apoptotic activities depending on the cell type and inflammatory context [44,45].

Defensins, the other major class of AMPs, are divided into α-defensins and β-defensins. α-defensins are primarily produced by neutrophils and play a crucial role in the initial immune response to infection [46]. β-defensins, on the other hand, are constitutively expressed on mucosal and dermal surfaces, providing a first line of defense against microbial invaders. Interestingly, osteoblasts, the cells responsible for bone formation, can also produce β-defensins in response to bacterial invasion. These small cationic peptides, particularly human β-defensin-1 (hBD-1) and human β-defensin-2 (hBD-2), interact with the negatively charged membranes of bacteria, disrupting their integrity and leading to their demise. β-defensins are expressed in both healthy and inflamed bone tissues, with hBD-2 being primarily upregulated in response to bacterial infection [11,47,48]. This observation suggests that AMPs are crucial in the host’s defense against osteomyelitis.

Generally, above described naturally occurring peptides exhibit potent killing activity against various microorganisms, including bacteria, fungi, and viruses. Importantly, AMPs can also target biofilms, which are a significant challenge in treating osteomyelitis, and they have a low propensity for inducing drug resistance in bacteria [43]. It is also worth emphasizing the immunomodulatory capacities of AMPs and their effect on enhancing bone healing, especially in the context of treatment of osteomyelitis [17].

## 6. AMPs in the Treatment of Bone Infections

### 6.1. Antimicrobial Peptides Versus Conventional Antibiotics

After debridement during the treatment of osteomyelitis, it is difficult to eradicate local microorganisms using conventional antibiotics. Furthermore, once bacterial cell clusters accumulate and adhere to a surface, biofilm formation significantly hinders antibiotic penetration and effectiveness. The prolonged local application of high-concentration antibiotics can lead to host cell toxicity, bone tissue damage, and increased bacterial resistance, ultimately hindering the healing process of affected bone. Therefore, developing biocompatible alternatives to antibiotics is crucial for effective bone infection treatment [43,49].

Currently, AMPs are regarded as a promising alternative to conventional antibiotics for treating bacterial infections. For instance, AMP PL-5 has been used to combat bacterial infections in the skin and wounds, particularly those caused by drug-resistant strains. Clinical trials have confirmed the safety and efficacy of the AMP PL-5 spray for treating skin wound infections [50]. Likewise, human lactoferrin N-terminal 11-peptide (hLF1-11) has demonstrated effectiveness in animal models of osteomyelitis and has shown promising results in phase I clinical trials [51].

### 6.2. Challenges and Prospects in Application of AMPs in Treatment of Osteomyelitis

As mentioned in the first part of this review, the treatment of osteomyelitis is challenging. Nearly 40% of clinical cases carry the risk of recurrence and chronic infection, which becomes particularly challenging to eliminate, especially when caused by drug-resistant microorganisms like methicillin-resistant *Staphylococcus aureus* (MRSA). This significantly increases treatment costs. The prevalence of chronic bone infections is rising due to the growing number of injuries from traffic accidents and implant-associated infections. An innovative approach to treating chronic osteomyelitis involves the local delivery of antimicrobials, ensuring high antibiotic concentrations at the infection site while minimizing systemic side effects [52,53].

Additionally, certain AMPs have been shown to support regenerative processes by promoting angiogenesis and osteogenesis, while simultaneously inhibiting osteoclastogenesis. Some AMPs could promote bone regeneration by modulating inflammatory response and enhancing osteogenesis by influence on stem cell behavior. Human LL-37 is an example of promising AMP with properties to promote the proliferation, migration, and osteogenic differentiation of MSCs [54].

Therefore, the potential of AMPs in treating bone infections extends beyond their direct antimicrobial activity. Research on AMP-loaded drug delivery systems has demonstrated their ability to reach infected sites, respond to local conditions, and maintain stability in the face of infection. In the context of osteomyelitis, ideal biomaterials exhibit antibacterial activity, enhance immunity, and provide a bioactive environment conducive to bone regeneration. For all these reasons, developing AMP-carrying biomaterials with multifunctional biological properties represents a significant advancement in this field [43,48].

Despite their broad-spectrum antimicrobial activity and regenerative properties, few cationic AMPs have progressed to clinical trials [17,43,55]. This is partly due to challenges associated with their systemic administration, including their short half-life in the body, susceptibility to protease degradation, and tendency to bind plasma proteins, which can reduce their biological activity. To overcome these limitations, researchers are exploring various strategies to enhance the stability and delivery of AMPs. These strategies include incorporating AMPs into biomaterials with specific properties, such as porosity, hydrophobicity, and electrostatic forces. Physical mixing and encapsulation of AMPs within biomaterials can improve their stability and bioactivity while minimizing cytotoxic effects. Encapsulation and controlled drug release efficiency depends on the specific material used [43,56].

AMPs can also self-assemble into nanostructures with other molecules, offering a novel approach to their delivery. Another effective method for modifying biomaterials with AMPs is coating, which creates surfaces resistant to bacterial contamination. Such coatings enhance AMP stability and reduce cytotoxicity at higher concentrations by evenly distributing peptides across the surface, ensuring good surface availability, and limiting protease-mediated degradation. Additionally, physical and chemical integration methods and cross-linking agents increase material-binding efficiency and reduce cytotoxicity [43,55].

## 7. Techniques for Immobilizing AMPs

Immobilizing AMPs onto biomaterials represents a cornerstone strategy for enhancing their therapeutic potential in combating osteomyelitis. This approach offers many benefits, including enhanced stability, controlled release, improved efficacy, and reduced toxicity. A diverse array of techniques has been developed to achieve effective AMP immobilization, each with advantages and limitations [43,57]. These techniques can be broadly categorized as follows:

Direct Immobilization

- Directed One-Step Immobilization: This streamlined approach involves directly attaching peptides to a material in a single step, often facilitated by chemical linkers that bridge the AMP and the biomaterial surface. For example, the G4S linker can connect hydroxyapatite (HAP) with LL-37, creating a potent weapon against Escherichia coli infections in bone tissue [58]. This technique is particularly attractive for its simplicity and efficiency, minimizing the required steps and reagents.

Covalent Immobilization

- Covalent Immobilization: This robust technique establishes a strong and stable chemical bond between the AMP and the biomaterial surface, ensuring that the AMPs remain securely attached and exert their therapeutic effects over an extended period. Examples include covalent binding of peptides like OP-145, Melimine, or KR-12 to materials like titanium, PLEXIGLAS, and PVC, creating durable antibacterial coatings effective against a broad spectrum of bacteria, including *S. aureus*, Pseudomonas aeruginosa, and Escherichia coli [59,60,61,62,63]. Covalent immobilization offers high stability and durability, making it suitable for applications where long-term AMP activity is desired [64].

Polymer-based Techniques

- Atom Transfer Radical Polymerization (ATRP): This sophisticated technique leverages the unique properties of polymers to achieve controlled and stimuli-responsive release of AMPs. For instance, the AMP HHC36, while potent against bacteria, can also cause cytotoxicity and delayed bone healing at high concentrations. HHC36 can be conjugated into a temperature-sensitive polymer, PNIPAM, to mitigate these adverse effects. This clever strategy allows for controlled peptide release at 25 °C before implantation, ensuring optimal integration with the host tissue. However, at 37 °C (body temperature), the HHC36 becomes concealed within the PNIPAM matrix, minimizing its interaction with host cells and reducing potential toxicity [65,66]. This approach exemplifies the potential of polymer-based techniques to fine-tune the release and activity of AMPs, optimizing their therapeutic efficacy and safety profile [54].

Surface Modification Techniques

- Surface Modification: This versatile approach involves altering the surface properties of the biomaterial to enhance AMP immobilization, bioactivity, and integration with the host tissue. For example, surface modification of titanium (Ti) through alkali treatment at room temperature creates a nanostructured network that enhances bone induction compared to pure Ti [67,68]. This modified surface can then be used to immobilize AMPs, such as lactoferrin (LF), which has been shown to increase osteogenic differentiation of rat bone marrow stromal cells (BMSCs), promoting bone regeneration and repair [69,70,71].
- Surface Coating: This widely used technique involves coating the biomaterial surface with a material layer that contains or binds to AMPs, creating a reservoir for sustained release and localized activity. For instance, methacryloyl gelatin (GelMA) combined with dopamine can modify HHC36 and silicate nanoparticles on the surface of hydrogels. The resulting composite material exhibits a remarkable combination of antibacterial and osteogenic properties. The HHC36 provides potent antibacterial activity against S. epidermidis and E. coli, while the silicate nanoparticles release magnesium, lithium, and orthosilicic acid ions, promoting bone formation and regeneration. This example highlights the potential of surface coating to create multifunctional biomaterials that address both the infection and the need for bone repair in osteomyelitis [69,72].

Electrochemical Techniques

- Anodic Oxidation and Covalent Linkage: This innovative technique combines the power of electrochemistry with the precision of covalent bonding to create a dynamic and responsive AMP delivery system. Anodic oxidation creates a porous surface on the biomaterial, increasing its surface area and creating sites for AMP attachment. Subsequently, covalent linkage is employed to securely attach AMPs to the modified surface. For example, anodic oxidation can be used to create TiO_2_ nanotubes, which can then be covalently bonded with polymers and AMPs, creating a pH-responsive system that releases AMPs as needed [73]. This intelligent design allows for a slow release of AMPs under physiological conditions, minimizing potential toxicity. In contrast, a rapid release is triggered in the acidic environment of an infection, providing a targeted therapeutic response [74].
- Anodic Oxidation and Electrodeposition: This powerful combination of electrochemical techniques allows for creating a robust and bioactive coating on the biomaterial surface. Anodic oxidation generates a porous oxide layer on the biomaterial, providing a scaffold for subsequent electrodeposition of AMPs. For instance, the CB peptide, known for its antibacterial activity, can be loaded with silk fibroin (SF) and electrodeposited onto a magnesium alloy (AZ31) that has undergone anodic oxidation [75]. This multifunctional coating not only improves the corrosion resistance of the alloy but also enhances its bioactivity, promoting bone cell adhesion and differentiation. The released CB peptide provides synergistic antibacterial effects, combating the infection and promoting bone healing [76,77].

Nanofiber-based Techniques

- Electrospinning: This versatile technique harnesses the power of an electric field to create nanofibers from a polymer solution, offering a unique platform for AMP immobilization and controlled release. These nanofibers can mimic the natural extracellular matrix, providing a supportive environment for cell adhesion and tissue regeneration. For example, gelatin and chitosan can be combined with PLGA microparticles loaded with AMP Pac-525 and hydroxyapatite nanoparticles (nHAP), creating a composite nanofiber scaffold with enhanced mechanical strength and osteogenic properties. Similarly, AMP GL13K and IDR-1018 can be incorporated into nanofibers formed by chitosan and oxidized pectin, creating a pH-responsive system that releases AMPs in response to the acidic environment of an infection. Nanofibers containing peptides like Tet213 and IDR-1018 showed efficacy against *S. aureus* and S. *oral* bacteria [78,79,80].
- Electrospraying: This technique utilizes an electric field to generate microparticles or nanoparticles from a liquid solution, offering a precise and controlled method for encapsulating and delivering AMPs. For instance, chitosan and PLGA can be combined to create microspheres that release AMP KSL-W in a controlled manner, effectively combating pathogens like *Fusobacterium nucleatum* and biofilms. This approach is particularly valuable for delivering AMPs that are susceptible to degradation or have short half-lives, as encapsulation within microparticles or nanoparticles can protect them and provide sustained release [79,81,82].

Other Techniques

- Physical Mixing and Coating: This straightforward approach involves physically mixing AMPs with the biomaterial or coating the biomaterial surface with AMPs, creating a simple and cost-effective method for AMP immobilization. For instance, chitosan–titanium implants can be coated with melittin, a potent AMP derived from bee venom, which synergizes with antibiotics to combat antibiotic-resistant *S. aureus*. This strategy highlights the potential of combining AMPs with conventional antibiotics to overcome antibiotic resistance and improve treatment outcomes [77,83,84].
- Grafting Technique: This technique involves chemically attaching AMPs to the biomaterial’s surface through a grafting process, creating a stable and durable bond. For example, chitosan membranes can be functionalized by grafting cysteine-modified AMP MSI-78 onto them, enhancing their antibacterial properties against bacteria like *S. epidermidis*. This approach can modify various biomaterials, tailoring their surface properties to enhance AMP immobilization and bioactivity [82].

**Figure 4:**
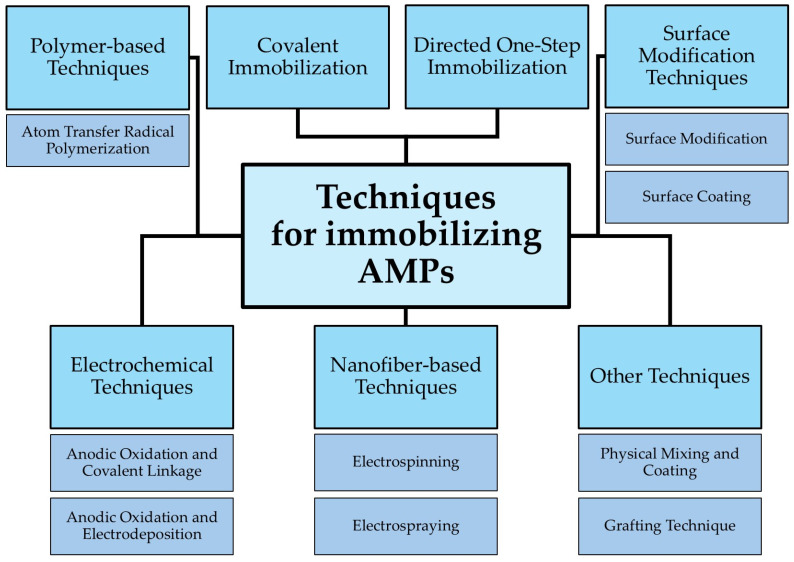
Techniques for Immobilizing AMPs.

The choice of immobilization technique depends on many factors, including the specific AMP being used, its physicochemical properties, the biomaterial chosen, the desired release profile, the target site, and the specific clinical application requirements. By carefully selecting and optimizing the immobilization strategy, researchers can harness the full therapeutic potential of AMPs, paving the way for more effective and innovative treatments for osteomyelitis. This ongoing research promises to revolutionize the management of this challenging bone infection, improving patient outcomes and reducing the burden of this debilitating disease [43,71,79,81]. Techniques for Immobilizing AMPs are summarized in Table 1 and Figure 4.

## 8. Animal Models for Human Study

**Figure 5:**
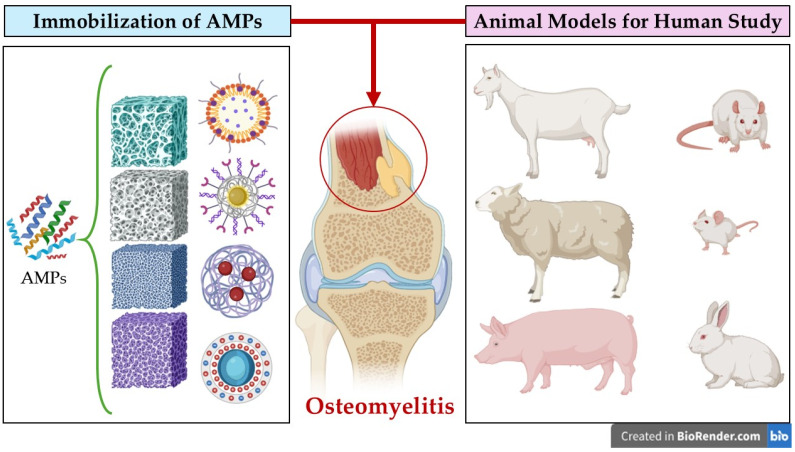
Diagram illustrating the application of antimicrobial peptides (AMPs) in osteomyelitis treatment and animal models used in translational research. On the left, different strategies for AMP immobilization in biomaterials are shown, while on the right, animal models commonly used for studying osteomyelitis therapies in humans are depicted.

Animal models are essential to assess the effectiveness of a newly introduced therapeutic agents and are widely reported in studies investigating the effectiveness of experimental AMPs-based agents in the treatment of osteomyelitis (Figure 5) [25,37,87]. 

### 8.1. The Importance of Animal Models in Studying Osteomyelitis

Animal models are indispensable tools in osteomyelitis research, providing valuable insights into disease pathogenesis, evaluating novel therapeutic approaches, and bridging the gap between preclinical studies and human clinical trials. Various animal species and models have been developed to mimic different aspects of human osteomyelitis, including fracture-associated infections, implant-related infections, and diabetic osteomyelitis [37,88].

#### 8.1.1. Rodent Models

Rodents, particularly mice and rats, are widely used due to their relatively low cost, ease of handling, well-characterized genetics, and availability of a wide range of research tools.

- Osteotomy Models: These models involve creating a surgical fracture (osteotomy) in the bone, often followed by inoculation with bacteria such as *S. aureus* or *S. epidermidis* [1]. They mimic fracture-associated osteomyelitis, allowing researchers to study the inflammatory response, bone healing, and the impact of different interventions. In a rat model of *Staphylococcus aureus*-induced bone infection following tibial osteotomy, increased levels of pro-inflammatory mediators (IL-1β, MIP-2) and the anti-inflammatory cytokine IL-10 were observed in the infected bone. Histological analysis revealed accumulations of immunocompetent cells and granulocytes at the osteotomy sites. Tartrate-resistant acid phosphatase (TRAP)-positive osteoclasts were rarely detected on the first postoperative day but became more prevalent by day 42, primarily in regions associated with bone damage and resorption. Other notable findings included periosteal reaction (new bone formation in response to injury), cortical thickening (increased bone density as a defense mechanism), and myeloid hyperplasia (expansion of bone marrow immune cells). These observations indicate that both pro-inflammatory and anti-inflammatory responses play a role in the progression of post-traumatic osteomyelitis [1,89].
- Mouse Models for Specific Virulence Factors: Mouse models have been used to investigate the role of specific *S. aureus* virulence factors in osteomyelitis. For instance, studies have shown that in Toxic Shock Syndrome Toxin 1 (TSST-1), a superantigen that triggers excessive immune activation, and protein A, a surface protein that facilitates immune evasion, both enhance bone resorption by activating osteoclasts. In contrast, PSM modulin toxins, which are small cytolytic peptides that disrupt host cell membranes, can induce bone loss through direct damage to osteoblasts [23,90].
- Diabetic Models: Diabetic mouse and rat models have been developed to study the impact of diabetes on osteomyelitis progression [91]. These models allow researchers to investigate the complex interplay between hyperglycemia, impaired immune function, and bacterial infection in osteomyelitis. These models are particularly relevant for studying osteomyelitis associated with diabetic foot infections, a serious complication in human patients [37,92].

#### 8.1.2. Large Animal Models

While rodent models offer many advantages, their small size can limit the ability to study certain aspects of osteomyelitis, such as implant-related infections and the use of clinically relevant surgical techniques. Larger animal models, such as rabbits, pigs, and sheep, are also used [37,93,94,95].

- Rabbit Models: Rabbits are commonly used due to their size, which allows for the testing of orthopedic device implantation and the evaluation of bone healing around implants. Their immune system is also more similar to that of humans than rodents, making them susceptible to infections [3]. Rabbits have been used to study the efficacy of various antimicrobial agents, biomaterials, and surgical techniques in treating osteomyelitis [95,96].
- Pig and Sheep/Goat Models: Pigs and sheep are also used, particularly for studies involving larger implants or those requiring more extensive surgical procedures [3]. These models are valuable for evaluating the biocompatibility and efficacy of new implant materials and designs and assessing the pharmacokinetics and pharmacodynamics of novel therapeutics [25,93,97].

### 8.2. Challenges and Considerations

While animal models offer invaluable tools for studying osteomyelitis, it is crucial to acknowledge their inherent limitations. Differences in immune responses and bone healing processes between animal species and humans can potentially influence the translatability of research findings. Additionally, variability within the same species, influenced by factors such as the specific model used and the bacterial strain, can introduce complexities in interpreting results. Ethical considerations surrounding the use of animals in research necessitate careful planning and adherence to strict guidelines to ensure humane treatment and minimize suffering [97,98].

Although animal models offer valuable insights, their limitations must also be considered [25,94,99]:

- Species Differences: The immune response and bone healing processes can differ between animals and humans, potentially affecting the translatability of findings [26,37].
- Model Variability: Even within the same species, there can be variability in the severity and progression of osteomyelitis depending on the specific model used, the bacterial strain, and other factors [25,97].
- Ethical Considerations: Using animals in research requires careful ethical consideration and adherence to strict guidelines to ensure humane treatment and minimize suffering [100].

### 8.3. Examples of AMPs Currently Tested on Animal Models

Christopher et al. confirmed the therapeutic efficacy of the antimicrobial peptide (AMP) hLF1-11 incorporated into Ca-P bone cement in a murine model of MRSA-induced osteomyelitis. Similarly, the AMP Dhvar-5 was incorporated into polymethylmethacrylate (PMMA) beads as a localized drug delivery system in a rabbit femur osteomyelitis model [51].

Alexandra et al. developed a topical drug delivery system that combines antimicrobial and regenerative properties for osteomyelitis treatment. This system comprised the AMP LL-18 (LLKKK18), vancomycin hydrochloride (VH), and an injectable oxydextrin (ODEX)-based hydrogel. In a murine model of MRSA-induced osteomyelitis, LL-18 demonstrated a dose-dependent immunomodulatory effect compared to 28 μM VH. A combination of 300 μM LL-18 and 483 μM VH successfully eradicated the infection in 70% of subjects, while also reducing tissue damage [101].

### 8.4. Future Directions

In osteomyelitis research, pursuing more sophisticated and human-relevant animal models is an ongoing endeavor. They include developing humanized mouse models, where human cells or tissues are engrafted into mice, creating a more human-like environment for studying the disease [102,103]. Another promising avenue is using 3D-printed bone models, which leverage advancements in 3D printing technology to create customized bone models that closely mimic human bone’s complex structure and composition. These models offer a unique opportunity to study the intricate interactions between bacteria, biomaterials, and host cells in a more realistic setting [104].

By judiciously selecting and refining animal models, researchers can continue to advance our understanding of osteomyelitis and pave the way for developing more effective treatments for this challenging condition.

## 9. Conclusions and Future Perspectives

Osteomyelitis presents a significant clinical challenge, particularly in open fractures and implant-associated infections. Conventional treatments, such as surgical debridement and systemic antibiotics, often prove inadequate due to the rise in antibiotic-resistant pathogens and the ability of bacteria to form biofilms. Antimicrobial peptides (AMPs), including LL-37 and β-defensins, represent a promising therapeutic alternative due to their broad-spectrum antimicrobial properties, low risk of resistance development, and ability to modulate the immune response while promoting wound healing.

Despite their immense therapeutic potential, several challenges must be addressed before AMPs can be widely applied in clinical practice. Optimizing efficacy is crucial, necessitating structural modifications to enhance antimicrobial activity against diverse pathogens and biofilms. Reducing cytotoxicity is another critical factor, requiring strategies to minimize toxicity to host cells while maintaining potent antimicrobial effects. Additionally, improving stability in the complex in vivo environment is essential for achieving sustained therapeutic outcomes. Developing effective delivery systems is also paramount; controlled-release systems are needed to ensure targeted delivery, prevent cytotoxic bursts, and maintain therapeutic AMP concentrations at the infection site. Furthermore, achieving multifunctionality is desirable, as AMP-based therapies should ideally combat infection while promoting bone regeneration. This can be accomplished by developing chimeric peptides with multiple functionalities or leveraging AMPs with inherent bone-regenerating capabilities, such as LL-37.

One of the most promising directions for AMP research is their integration into biomaterials for bone tissue engineering. Novel immobilization techniques and innovative biomaterial designs have been explored to enhance AMP effectiveness, ensuring a sustained antimicrobial effect while supporting tissue regeneration. However, significant discrepancies often arise between in vitro and in vivo studies, potentially leading to increased toxicity, reduced stability, or lower-than-expected therapeutic efficacy. Therefore, future research should prioritize in vivo studies to assess the therapeutic potential of novel AMP-based biomaterials under realistic physiological conditions.

Animal models provide a controlled and reproducible environment that can be adapted to mimic different disease conditions more accurately than data obtained from clinical cases. Their role should be emphasized as a valuable tool for understanding the pathogenesis of osteomyelitis and for developing innovative therapies that not only combat infections but also promote bone healing. Translational research efforts should focus on bridging the gap between preclinical and clinical studies, ensuring that AMP-based therapies are both safe and effective before they reach widespread clinical application.

By addressing these challenges, AMP-based therapies could revolutionize osteomyelitis treatment, providing an innovative alternative to conventional approaches. Continued research and development in this field hold the potential to redefine the management of bone infections, offering more effective, targeted, and resistance-free therapeutic options for patients facing this debilitating condition.

## Figures and Tables

**Table 1:** Techniques for immobilizing AMPs and targeting bacteria.

Immobilization Technique	AMPs	Material	Target Bacteria	Ref.
Directed One-Step Immobilization	LL-37	HAP	*Escherichia coli*	[58]
Covalent Immobilization	KR-12	Ti, PLEX, PVC, PEEK	*Staphylococcus aureus*, *MRSA*, *S. epidermidis*, *MRSE*, *E. coli*	[59,60,61,62,63]
Atom Transfer Radical Polymerization (ATRP)	HHC36	PNIPAM	*Staphylococcus aureus*	[65,66]
Surface Modification	HHC36	Ti, collagen (NCS)	*Escherichia coli*, *S. aureus*	[67,68,69,70,71]
Chemical Integration	HHC36	Ti	*MRSA*, *E. coli*, *Pseudomonas aeruginosa*	[85]
Surface Coating	HHC36	GelMA-DOPA	*E. coli*, *S. aureus*, *P. aeruginosa*, *S. epidermidis*	[72]
Anodic Oxidation and Covalent Linkage	HHC36	PMAA/Ti	*E. coli*, *S. aureus*, *Pseudomonas aeruginosa*, *MRSA*	[73]
Anodic Oxidation and Electrodeposition	CB	AZ31	*Escherichia coli*	[75]
Electrostatic Spinning	Tet213, IDR-1018	CS, Ti	*Oral Streptococci*, *Staphylococcus aureus*	[78,80]
Electrospray Technology	KSL-W	PLGA/CS	*Fusobacterium nucleatum*	[86]
Physical Coating	Melittin	CS-Ti	*Staphylococcus aureus*	[84]
Grafting Technique	MSI-78	CS	*Staphylococcus epidermidis*	[82]

## Data Availability

No new data were created or analyzed in this study. Data sharing is not applicable to this article.
